# Label-free drug discovery

**DOI:** 10.3389/fphar.2014.00052

**Published:** 2014-03-27

**Authors:** Ye Fang

**Affiliations:** Biochemical Technologies, Science and Technology Division, Corning IncorporatedCorning, NY, USA

**Keywords:** cell phenotypic screen, drug safety/toxicity, label-free drug discovery, lead selection, molecular mechanism of action, phenotypic screen, polypharmacology, target identification

## Abstract

Current drug discovery is dominated by label-dependent molecular approaches, which screen drugs in the context of a predefined and target-based hypothesis *in vitro*. Given that target-based discovery has not transformed the industry, phenotypic screen that identifies drugs based on a specific phenotype of cells, tissues, or animals has gained renewed interest. However, owing to the intrinsic complexity in drug–target interactions, there is often a significant gap between the phenotype screened and the ultimate molecular mechanism of action sought. This paper presents a label-free strategy for early drug discovery. This strategy combines label-free cell phenotypic profiling with computational approaches, and holds promise to bridge the gap by offering a kinetic and holistic representation of the functional consequences of drugs in disease relevant cells that is amenable to mechanistic deconvolution.

## INTRODUCTION

Early drug discovery is achieved mainly through two strategies, target-based and phenotypic approaches (Hart, 2005; Swinney and Anthony, 2011). Target-based screens use high-throughput and label-dependent molecular assays to measure the effect of compounds on a specific target protein *in vitro*, while phenotypic screen use unbiased phenotypic assays to examine the effect of compounds on a specific phenotype of cells, tissues or animals. Target-based approaches have been dominating early drug discovery in the past quarter of century, which is coincident with the continuous decline in productivity of pharmaceutical research and development (Paul et al., 2010; Pammolli et al., 2011; Scannell, 2012). Several factors contribute to this productivity crisis. First, there have been increasing efforts in high-risk projects for unmet therapeutic needs and associated with unexploited biological mechanisms in the past decades (Kamb et al., 2007; Hopkins, 2008; Rask-Andersen et al., 2011). Second, the target chosen in a screen may be not essential to disease pathogenesis or induce undesired toxicity, and the molecular mechanism of action (MMOA) investigated may be unable to produce therapeutic benefits (Hopkins, 2008; Swinney and Anthony, 2011). The MMOA describes the interaction between a drug and its target (or targets) that creates a specific response. Third, many, if not all, drugs display clinically relevant polypharmacology – the specific binding of a drug to more than one target (Roth et al., 2004; Yildirim et al., 2007; Hopkins, 2008; Rask-Andersen et al., 2011), suggesting that single target-based screen may *de facto* be ineffective. Fourth, molecular assays for target-based screens generally rely on the use of labels, which may cause artifacts in results (Beher et al., 2009; Pacholec et al., 2010; Hu et al., 2012). Lastly, traditional phenotypic approaches suffer disadvantages associated with low-to-moderate throughput, and difficulty in target deconvolution and in governing medicinal chemistry optimization (Kenakin, 2009; Swinney and Anthony, 2011).

In the past years, phenotypic screens have gained renewed interest in discovering first-in-class or best-in-class medicines (Lee et al., 2012; Eggert, 2013). Comparing to traditional phenotypic approaches, label-free cell phenotypic profiling techniques afforded by optical or electric biosensors offer clear advantages in rich information content, real-time kinetics, highly flexible assay formats, and high-throughput, beside wide pathway coverage and ability in multi-target profiling and screening that are common to all phenotypic assays (Fang, 2013). Optical biosensors such as resonant waveguide grating (RWG) measure drug-induced dynamic mass redistribution (DMR) signals, while electric biosensors measure drug-induced impedance signals (Fang, 2010). In parallel, similarity analysis based on two-dimensional structures of compounds has been used to predict drug–target interactions (Keiser et al., 2007, 2009; Lounkine et al., 2012), while molecular docking using ever increasing numbers of three-dimensional protein structures are also productive (Carlsson et al., 2011; Koutsoukas et al., 2011; Shoichet and Kobilka, 2012; Stevens et al., 2012).

**FIGURE 1:**
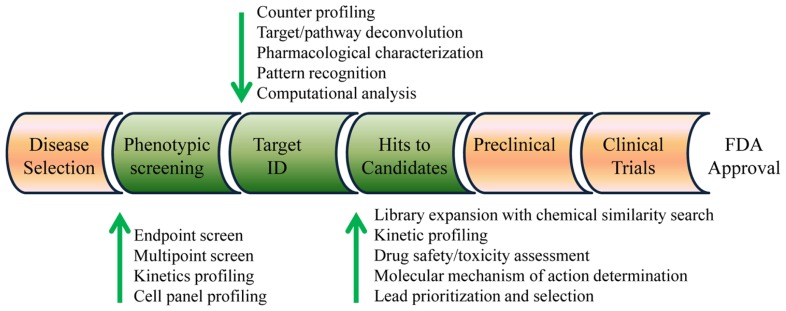
**Label-free drug discovery strategy.** Combining computational approaches with label-free cell phenotypic profiling and screening techniques can be used for high-throughput screening, target engagement determination, compound library expansion, lead optimization, molecular mechanism of action determination, drug safety/toxicity assessment, and lead prioritization and selection.

Herein, I propose a label-free strategy combining label-free cell phenotypic profiling techniques with computational approaches for early drug discovery (**Figure 1**). Essential to this strategy is that label-free cell phenotypic profiling techniques are used for multi-target screening, target identification, MMOA determination, and lead selection. Bioinformatics analysis of the label-free profiles of compounds is used to provide analytical support for target identification, and chemical similarity analysis is used to expand compound library for lead optimization and selection. Of note, the principles and applications of label-free biosensors for cell analysis have been widely reviewed in literature (Fang, 2006,2011b; McGuinness, 2007), and thus not included in the present review.

## LABEL-FREE CELL PHENOTYPIC SCREENING

### CHOICE OF CELLS

As the basic unit of life cells have been widely used for drug discovery, mostly because the functional responses of drugs in cells provide better understanding of receptor physiology and drug pharmacology than *in vitro* binding studies. Target-based approaches often use recombinant cell lines expressing a specific target implicated in a disease, while cell phenotypic approaches often use native cells including immortalized cell lines, primary cells, and stem cells. As surface sensitive and non-invasive techniques label-free biosensors can examine drug-induced minute changes in a confluent layer of eventually all types of cells (Fang, 2010, 2011a), including primary (Hennen et al., 2013) or stem cells (Bagnaninchi and Drummond, 2011; Abassi et al., 2012; Pai et al., 2012). Compared to recombinant cell lines, primary or stem cells retain many functions seen *in vivo* and express endogenous targets of interest in their native signaling circuitry, thus permitting drug profiling using more physiologically and clinically relevant cell phenotypes (Kenakin, 2009; Eglen and Reisine, 2011). Owing to its spatially resolved capability the recently developed RWG imager enables drug profiling using partially confluent cells (Ferrie et al., 2010) or even single cells (Ferrie et al., 2012), and thus opens an unique opportunity to screen drugs using primary or stem cells when homogeneous cell populations are difficult to obtain (Pai et al., 2012).

### CHOICE OF CELLULAR PHENOTYPES

**FIGURE 2:**
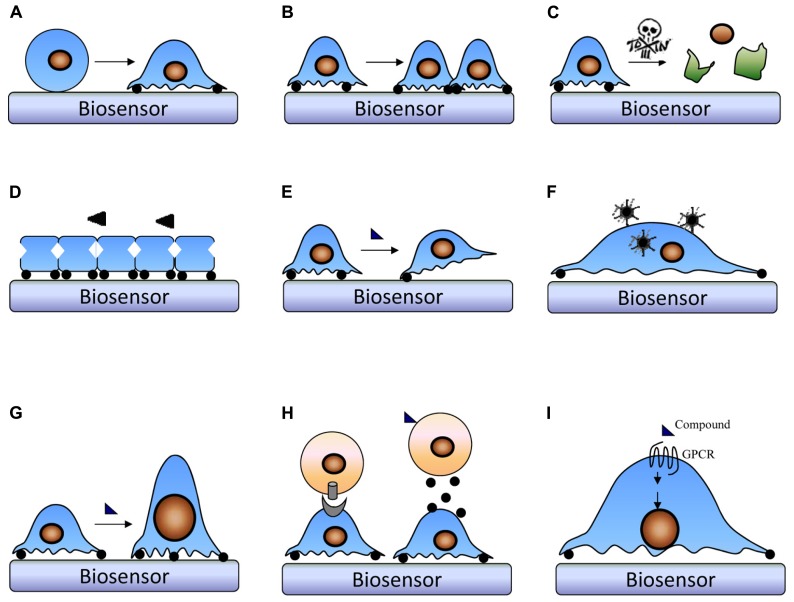
**Representative label-free cellular phenotypes examined with label-free techniques.** Label-free biosensors can be used to monitor in real-time a great number of cellular process ranging from cell adhesion **(A)** to cell proliferation **(B)**, cell death **(C)**, cell barrier function **(D)**, cell migration **(E)**, viral infection **(F)**, cell morphology **(G)**, cell–cell communication **(H)**, and cell signaling **(I)**. To monitor different cellular phenotypes, different assay conditions may be applied.

Disease relevant cellular phenotypes can be structural, morphological, or physiological abnormalities involving cells or cell components. Structural abnormalities can be classified based on cellular component hierarchy, whereas abnormal morphology phenotypes is either the (abnormal) absence of required cellular parts, the (abnormal) presence of additional cellular parts, or abnormal qualities of cellular parts, and abnormal physiology of a cell component refers to abnormal functionality of a cell component (Hoehndorf et al., 2012). Thus, drug profiling and screening can be performed using a great number of cellular phenotypes such as angiogenesis, cell death, cell division, and inflammation; depending on the MMOA of interest one or more specific cellular phenotype may be examined (Welsh et al., 2009; Kepp et al., 2011). For label-free cell phenotypic screening, two common approaches developed are endpoint and kinetic based screens (see below). Given that label-free biosensors are sensitive to cell numbers, cell signaling and morphological changes, these biosensors permit screening and profiling compounds in the context of a great number of cellular phenotypes ranging from cell adhesion to cell life cycle (cell cycle progression, division, and growth), receptor signaling, cell death, viral infection, cell migration and invasion, and cell-cell communication (**Figure 2**).

### ENDPOINT HIGH-THROUGHPUT SCREENS

The endpoint screens leverage the ability of a biosensor to record and encode the signaling events of a specific receptor in a population of cells (typically confluent cells) into an integrated and kinetic biosensor response for identifying active molecules specific to the receptor (Fang, 2010). Here, once the biosensor profile of a receptor cognate agonist in the cells is obtained, its response at a specific time point is monitored and used as the readout to fish out ligands for the receptor of interest from a compound library. In order to identify distinct classes of ligands screening can be performed using different formats. For instance, one-step assay may be useful for discovering agonists, wherein the cells are stimulated with compounds, each individually. Considering the wide presence of compensatory pathways in cell signaling, the one-step agonist screen may result in relatively high false positives for the receptor of interest. Such false positives can be minimized using two-step endpoint screens, wherein the cells are stimulated with compounds first, followed by stimulation with a cognate agonist specific to the receptor. Compounds that are active in the first step and also desensitize the second agonist stimulation would be agonists for the receptor, while compounds that are inactive in the first step but block the second agonist stimulation would be antagonists for the receptor (Tran and Fang, 2008; Deng et al., 2011a). In addition, a three-step assay wherein a compound washout step is introduced between compound and receptor cognate agonist stimulation steps can be useful for identifying long-acting antagonists or agonists (Goral et al., 2011; Deng et al., 2012b).

### MULTI-PARAMETER SCREENS

Receptor signaling is encoded by the coupling of temporal dynamics with spatial gradients of signaling activities (Kholodenko, 2006), and may come in multiple pathways and waves (Ferrie et al., 2013; Lohse and Calebiro, 2013). Consequently, label-free biosensors as a non-invasive recorder mirror the dynamics of receptor signaling, and the biosensor signature arising from the activation of a receptor could contain multiple phases. Therefore, multi-parameter profiling and screening may be feasible and offer additional information regarding to the specificity and mechanisms of action of hits to a receptor, a signaling protein, or a pathway.

### REAL-TIME KINETIC PROFILING

**FIGURE 3:**
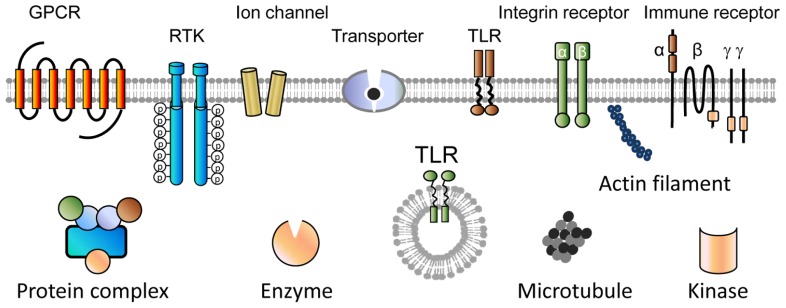
**Target receptor classes whose activation or modulation has been shown to trigger characteristic biosensor responses in living cells.** Label-free receptor signaling profiling has wide coverage in targets and pathways. GPCR, G protein-coupled receptor; RTK, receptor tyrosine kinase; TLR, Toll-like receptor.

For label-free cell phenotypic screens, real-time kinetic measurements of drug action would be more informative but with lower throughput than end-point screens. The kinetic profiling of compounds may be performed in the context of a specific cellular process such as cell adhesion, cell growth and death, or cell signaling. Label-free biosensors allow for interrogating drug molecules with wide coverage in targets and pathways of native cells (**Figure 3**; Fang, 2011b, 2013). The modulation of many classes of targets including G protein-coupled receptors (GPCRs), receptor tyrosine Kinases (RTKs), transporters, Toll-like receptors (TLRs), immune receptors, enzymes, cell structural proteins, and kinases can directly lead to rapid biosensor responses. The most popular is to profile compound-induced cell signaling in confluent cells, given that the cells once reach confluency start to enter a new growth cycle or a quiescent state and the compound-induced response is almost exclusively due to cell signaling (Fang, 2010, 2011a). Alternatively, the long-term impacts of compounds on cell growth can also be used to screen compound library (Abassi et al., 2009; Fu et al., 2011). Of note, this approach may be able to identify ligands for other classes of targets such as nuclear receptors whose activation by themselves may not result in rapid signaling-related biosensor responses.

### CELL PANEL PROFILING AND SCREENING

Large panels of disease relevant cell lines annotated with both genetic and pharmacological data are powerful tools for drug discovery (Barretina et al., 2012; Garnett et al., 2012). For instance, NCI60 consists of 60 (now 59) human cancer cell lines from nine different tissues introduced in 1990 by the US National Cancer Institute (NCI) in Bethesda, Maryland, and has been widely used for discovering new anticancer drugs (Shoemaker, 2006). For label-free profiling, cell panels may consist of multiple cell lines for a disease, a parental cell line and its recombinant counterparts, or a stem cell and its differentiated cells. Each cell line has unique expression pattern of functional receptors and signaling circuitry. Thus, the use of cell panels not only expands the number of addressable targets/pathways, but also offers confirmative information regarding to the potential mechanism of action of active compounds identified in label-free cell phenotypic screens (Verdonk et al., 2006; Morse et al., 2011, 2013; Pai et al., 2012; Ferrie et al., 2014). For instance, using the DMR assay we profiled a library of sixty-nine ligands of adrenergic receptor (AR) with a cell panel consisting of the parental HEK-293 and four β_2_-AR-stably expressed cell lines, and found that HEK-293 endogenously expresses functional G_i_-coupled α_2_-AR and G_s_-coupled β_2_-AR, and these ligands displayed divergent label-free cell phenotypic pharmacology (Ferrie et al., 2014).

## TARGET IDENTIFICATION AND VALIDATION

Identifying the target for a specific phenotype is vital to guide lead optimization and to understand the potential toxicity for the target (Hart, 2005). Common to classic phenotypic approaches for determining target engagement is to first generate target hypotheses using pattern recognition to compare small molecule phenotypic profiles to those of known reference molecules, followed by confirmation using direct proteomic approaches (Young et al., 2008; Schenone et al., 2013). However, classical phenotypic approaches mostly rely on descriptive, empirical, and end-point measurements, which, by themselves, generally offer little insights about the biological mechanisms of action of drugs (Feng et al., 2009). In contrast, label-free cell phenotypic approaches all measure real-time kinetic responses of compounds in cells, which contain target- and pathway-specific information (Fang, 2011a). For label-free endpoint and multi-parameter screens, target hypothesis is predefined by the reference agonist cognate to the receptor of interest, so target engagement can be confirmed using direct binding assays or counter profiling using another cell line that does not express the target receptor. For instance, using the DMR signal at 25 min post-stimulation with methacholine in CHO-M_3_ cells as the readout, screening a library of 83,000 compounds led to identification of 49 novel muscarinic M_3_ receptor ligands that had pIC_50_ values between 4.8 and 6.3 and were further confirmed using radiobinding assays (Dodgson et al., 2009). Here, methacholine is used as the reference agonist for the M_3_ receptor. Of note, these novel ligands were found to be false negatives in a calcium flux assay.

For label-free kinetic profiling, target hypothesis can be generated using several approaches. First, target/pathway deconvolution may be directly achieved through investigating the impact of chemical probes and/or genetic manipulations (e.g., RNAi) on the kinetic response of a compound itself (Fang et al., 2005; Deng et al., 2011b; Verrier et al., 2011). Second, counter profiling between a recombinant cell line expressing the receptor of interest and its parental cell line without the receptor is also effective to confirm the target specificity (Ferrie et al., 2014). Third, multiple assays including agonist, antagonist, desensitization, and antagonist reversal assays when respective pharmacological tools are available can be used to ascertain the specificity of a compound to the receptor of interest (Ferrie et al., 2013). Fourth, pattern recognition based on label-free profiles of compounds can be used to generate target hypothesis through comparison of their profiles with databases of the activity profiles of other reference molecules with known targets (Abassi et al., 2009; Fu et al., 2011). Traditional approaches including proteomics-, genetics-, and bioinformatics-based approaches can then be used for determining target engagement (Ziegler et al., 2013). Fifth, computational approaches based on similarity analysis of known probe molecules (Keiser et al., 2007, 2009; Lounkine et al., 2012) or molecular docking (Carlsson et al., 2011; Shoichet and Kobilka, 2012) can be used to predict the probability of the binding of small molecules to a specific target.

## HIT IDENTIFICATION

For label-free endpoint screens, hits are selected based on the label-free profile arising from the activation of the receptor of interest, similar to classical target-based screens (Dodgson et al., 2009). For label-free kinetic profiles, hits are selected based on a specific phenotypic response in the context of a specific cellular process, such as the increase in label-free profile of cell adhesion, alteration of the label-free profile of cell growth, or a specific label-free profile of cell signaling. For instance, screening a library of 660 compounds led to identification a characteristic DMR signal in HT-29 cells for a subset of compounds (Deng et al., 2011b). Combining DMR antagonist/desensitization assays with GPR35 knockdown with interference RNA, receptor internalization, and Tango β-arrestin translocation assays revealed that two novel series of chemical compounds, 2-(4-methylfuran-2(5H)-ylidene)malononitrile and thieno[3,2-b]-thiophene-2-carboxylic acid derivatives, are GPR35 agonists.

## LEAD OPTIMIZATION

Once hits are identified and confirmed, searching similar compounds from commercial and public databases can quickly expand compound library for generating structure-activity relationship (SAR) analysis. These databases include PubChem (Wang et al., 2009), ChemBank (Seiler et al., 2008), DrugBank (Knox et al., 2011), ChemBL (Gaulton et al., 2012), and ZINC (Irwin and Shoichet, 2005). With the ever-increasing number of compounds annotated with biological and pharmacological activities in these databases, it is highly possible to quickly identify lead-like compounds with high specificity and potency to the target receptor. For instance, according to the similarity of tyrphostins to 2-(4-methylfuran-2(5H)-ylidene)malononitrile compounds, we hypothesized and confirmed that a group of tyrphostins such as tyrphostin-51 are GPR35 agonists with moderate potency (Deng et al., 2011a). Given that tyrphostins, the first generation of tyrosine kinase inhibitors, are tyrosine analogs (Levitzki and Mishani, 2006), we hypothesized and confirmed that multiple tyrosine metabolites are GPR35 agonists (Deng and Fang, 2012b; Deng et al., 2012a). From these SAR studies, we further expanded the chemical library by searching public databases and identified a group of nitrophenols as GPR35 agonists, among which 4,4’-(2,2-dichloroethene-1,1-diyl)bis(2,6-dinitrophenol) displays high potency with an EC_50_ of 6nM (Deng and Fang, 2012a).

## DRUG SAFETY/TOXICITY ASSESSMENT

Drug toxicity/safety assessment is essential to drug discovery and development, and may be studied using several label-free cell phenotypic profiling approaches. First, the recently developed high frequency electric impedance biosensor system can be used to monitor the impact of drugs on the beating patterns of primary or stem cell-derived cardiomyocytes; and this system can recapitulate known effects of various known modulators of cardiac function (Abassi et al., 2012). Cardiac toxicity is one of the major concerns in drug development, and accounts for one-third of all drug withdrawals from the market (Wilke et al., 2007). Second, potential adverse drug reactions (ADRs) of compounds can be assessed using a panel of cells consisting a parental cell line and a number of recombinant cell lines, each expressing a specific target receptor that is known to be associated with and prone to cause ADRs. ADRs are the second leading cause for attrition of drug candidates in clinical trials, behind lack of efficacy (Arrowsmith, 2011). Factors that cause ADRs include the primary target of the drug itself, non-specific interactions of reactive metabolites of the drug, or unintended activity at off-targets. The number of off-targets that is known to be associated with ADRs is relatively small (~75; Bender et al., 2007; Campillos et al., 2008; Lounkine et al., 2012), almost all of which can be directly examined using label-free profiling. Practically, these recombinant cell lines can be made readily to be profiled as cell bank (e.g., frozen cell batches), or transitly transfected *in situ* using classical viral or plasmid DNA-based approaches. Third, computational approaches based on chemical structures or molecular docking can be used to calculate the probability of drug candidate molecules binding to these ADR-related targets (Lounkine et al., 2012).

## MMOA DETERMINATION

Elucidating the MMOA of drug candidate molecules is a critical step in drug discovery. Label-free biosensor such as surface plasmon resonance and RWG is well-known for its ability to determine the affinity and kinetics of drugs binding to their primary target (Schuck, 1997; Fang, 2012). Label-free cell phenotypic profiling also can provide information regarding to the MMOA of compounds. This is done through leveraging the sensitivity of the label-free profiles of compounds to their polypharmacology (Wermuth, 2004), functional selectivity (or biased agonism; Kenakin, 2012), binding kinetics (Deng et al., 2013a), binding orientation (Bock et al., 2014), cell membrane permeability (Ferrie et al., 2013), and transport mechanisms (Deng et al., 2013b; reviewed in Fang, 2013).

The biased agonism describes ligand-dependent selectivity for a specific signal transduction pathway over others downstream the same receptor, and is common to ligands for GPCRs and potentially other classes of receptors (Fang, 2013). Owing to its integrative nature in measurement (Fang et al., 2006), label-free cell phenotypic profiling, by *de facto*, is not ideal for directly assessing biased agonism (Morse et al., 2013). However, several label-free approaches may be useful to manifest biased agonism. First, multi-parameter kinetic analysis may sort ligands for a specific target into different clusters. For instance, profiling of a set of β_2_-adrenergic receptor (β_2_-AR) ligands in A431 cells using DMR assays revealed that multiple kinetic parameters extracted from their responses allow fine classification of these ligands based on their efficacy and biased agonism (Fang and Ferrie, 2008; Fang, 2010). Second, the recent developed integrative pharmacology ontarget (iPOT) approach can classify ligands based on their specificity, pathway selectivity, and efficacy for the target receptor of interest (Ferrie et al., 2011; Morse et al., 2011, 2013). The iPOT approach leverages distinct sensitivity of the label-free profiles of different drugs acting through the same receptor in different cell lines, or the same cell line but with different preconditioning. The cell preconditioning can be achieved using specific probe molecules to impair or alter specific pathways, or genetic tools to alter the expression of a specific signaling protein. Using this approach, we had obtained a pharmacological heatmap of all adrenergic receptor drugs approved by the US Food and Drug Administration that correlates well with their *in vivo* indications (Ferrie et al., 2011).

The functional consequence of different binding kinetics of a family of ligands for a specific receptor can also be assessed using label-free biosensor ligand washout assay. The onboard microfluidics is the most effective means to control the duration of a ligand exposed to the cells, so it is possible to determine whether the effect of the ligand is short or long acting. For instance, using microfluidics to control the agonist stimulation duration we found that the activation of the β_2_-AR in A431 with a pulse of its agonist as short as 1 min is sufficient to trigger a sustained response, whose sustainability is dependent on the type and concentration of agonists, and the stimulation duration (Goral et al., 2011; Ferrie et al., 2013). In another study, combining radiobinding results with electric biosensor profiling results revealed that the efficacy of adenosine A_2A_ receptor agonists is positively correlated to their receptor residence time (the reciprocal of off rate; Guo et al., 2012). Almost identical trend was found for a family of agonists for endogenous muscarinic M_3_ receptors in six different cell lines (Deng et al., 2013a).

The cell membrane permeability and transport mechanism are important mostly for the efficacy of drugs acting at intracellular targets. A recent DMR study of three inhibitors for epidermal growth factor receptor (EGFR) in A431 and HT-29 showed that the recovery of EGFR signaling after inhibitor removal from the extracellular buffer was faster in HT-29 than in A431, and also dependent on the duration of inhibitor removal (Deng et al., 2013b). Furthermore, the potency of three inhibitors including gefitinib, erlotinib, and AG1478 was generally higher in A431 than HT-29 cells. The most possible mechanism for this is that the drug uptake and retention paly a dominating role in determining the whole cell efficacy of these kinase inhibitors. The cellular retention of these inhibitors is a function of cell uptake and effluxing via efflux transporter such as breast cancer resistance protein (BCRP/ABCG2). All three inhibitors tested are ABCG2 substrates; A431 cells express little ABCG2, while HT-29 expresses high amount of ABCG2.

## LEAD SELECTION AND PRIORITIZATION

Effective lead selection and prioritization is essential for getting the cost of early drug discovery under control. In a typical screening campaign, tens of thousands of hits are often identified. After optimization, about one hundred lead-like molecules are selected for animal testing. The iPOT approach, label-free profiling techniques in general, are useful to classify these lead-like molecules into distinct clusters, each of which may share a common MMOA (Ferrie et al., 2011). Representative lead-like molecules from each cluster can then be selected for *in vivo* testing (Morse et al., 2013). Computational approaches, in particular chemical similarity analysis against the ADR-related receptor panel, would also be beneficial to lead selection process.

## CONCLUSION

In the recent years, there has been a renaissance in phenotypic approaches for drug discovery. Label-free cell phenotypic profiling and screening holds great promise in discovering disease-modifying activities of drug molecules via validated or previously undescribed targets, or by acting simultaneously on more than one target. Combining computational approaches, in particular similarity analysis based on chemical structures and molecular docking based on three-dimensional structures of target proteins, with label-free approaches would greatly facilitate early drug discovery by permitting target engagement determination, compound library expansion, MMOA deconvolution, safety and toxicity assessment, and lead optimization and selection.

## Conflict of Interest Statement

Ye Fang is a research director/fellow of Corning Incorporated. DMR technology is patented by Corning Incorporated.
